# Metabolomics and systems pharmacology: why and how to model the human metabolic network for drug discovery^☆^

**DOI:** 10.1016/j.drudis.2013.07.014

**Published:** 2014-02

**Authors:** Douglas B. Kell, Royston Goodacre

**Affiliations:** School of Chemistry and Manchester Institute of Biotechnology, The University of Manchester, 131 Princess Street, Manchester M1 7DN, UK

## Abstract

Metabolism represents the ‘sharp end’ of systems biology, because changes in metabolite concentrations are necessarily amplified relative to changes in the transcriptome, proteome and enzyme activities, which can be modulated by drugs. To understand such behaviour, we therefore need (and increasingly have) reliable consensus (community) models of the human metabolic network that include the important transporters. Small molecule ‘drug’ transporters are in fact metabolite transporters, because drugs bear structural similarities to metabolites known from the network reconstructions and from measurements of the metabolome. Recon2 represents the present state-of-the-art human metabolic network reconstruction; it can predict *inter alia*: (i) the effects of inborn errors of metabolism; (ii) which metabolites are exometabolites, and (iii) how metabolism varies between tissues and cellular compartments. However, even these qualitative network models are not yet complete. As our understanding improves so do we recognise more clearly the need for a systems (poly)pharmacology.

## Highlights

• We now have metabolic network models; the metabolome is represented by their nodes.
• Metabolite levels are sensitive to changes in enzyme activities.
• Drugs hitchhike on metabolite transporters to get into and out of cells.
• The consensus network Recon2 represents the present state of the art, and has predictive power.
• Constraint-based modelling relates network structure to metabolic fluxes.

## Introduction – a systems biology approach to drug discovery

**Figure 1:**
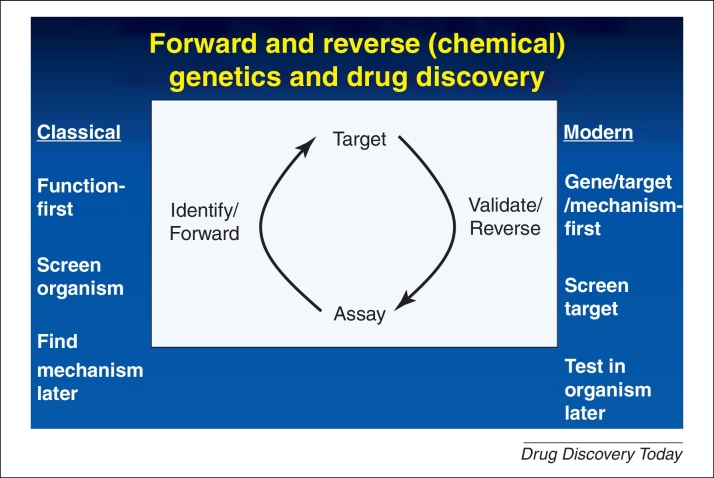
The change in drug discovery strategy from ‘classical’ function-first approaches (in which the assay of drug function was at the tissue or organism level), with mechanistic studies potentially coming later, to more-recent target-based approaches where initial assays usually involve assessing the interactions of drugs with specified (and often cloned, recombinant) proteins *in vitro*. In the latter cases, effects *in vivo* are assessed later, with concomitantly high levels of attrition.

It is clearly not news that the productivity of the pharmaceutical industry has declined significantly during recent years [1–14] following an ‘inverse Moore's Law’, Eroom's Law [11], or that many commentators, for example, see [7,8,14–47], consider that the main cause of this is because of an excessive focus on individual molecular target discovery rather than a more sensible strategy based on a systems-level approach (Fig. 1).

**Figure 2:**
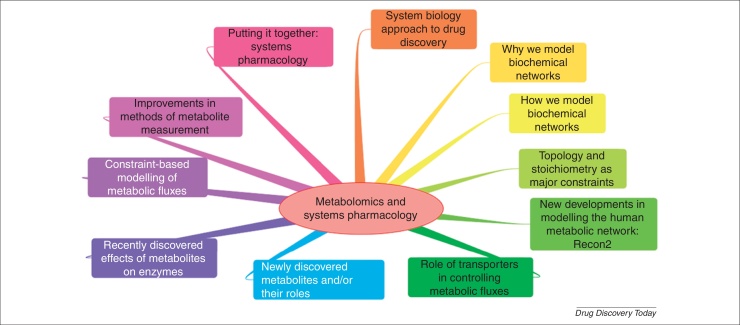
A Mind Map summarising this paper.

Arguably the two chief hallmarks of the systems biology approach are: (i) that we seek to make mathematical models of our systems iteratively or in parallel with well-designed ‘wet’ experiments, and (ii) that we do not necessarily start with a hypothesis [48,49] but measure as many things as possible (the ’omes) and let the data tell us the hypothesis that best fits and describes them. Although metabolism was once seen as something of a Cinderella subject [50,51], there are fundamental reasons to do with the organisation of biochemical networks as to why the metabol(om)ic level – now in fact seen as the ‘apogee’ of the ’omics trilogy [52] – is indeed likely to be far more discriminating than are changes in the transcriptome or proteome. The next two subsections deal with these points and Fig. 2 summarises the paper in the form of a Mind Map.

## Modelling biochemical networks – why we do so

As set out previously [19,53–55], and as can be seen in every systems biology textbook [56–58], there are at least four types of reasons as to why one would wish to model a biochemical network:

• Assessing whether the model is accurate, in the sense that it reflects – or can be made to reflect – known experimental facts.
• Establishing what changes in the model would improve the consistency of its behaviour with experimental observations and improved predictability, such as with respect to metabolite concentrations or fluxes.
• Analyzing the model, typically by some form of sensitivity analysis [59], to understand which parts of the system contribute most to some desired functional properties of interest.
• Hypothesis generation and testing, enabling one to analyse rapidly the effects of manipulating experimental conditions in the model without having to perform complex and costly experiments (or to restrict the number that are performed).

In particular, it is normally considerably cheaper to perform studies of metabolic networks *in silico* before trying a smaller number of possibilities experimentally; indeed for combinatorial reasons it is often the only approach possible [60,61]. Although our focus here is on drug discovery, similar principles apply to the modification of biochemical networks for purposes of ‘industrial’ or ‘white’ biotechnology [62–68].

Why we choose to model metabolic networks more than transcriptomic or proteomic networks comes from the recognition – made particularly clear by workers in the field of metabolic control analysis [69–77] – that, although changes in the activities of individual enzymes tend to have rather small effects on metabolic fluxes, they can and do have very large effects on metabolite concentrations (i.e. the metabolome) [78–81]. Thus, the metabolome serves to amplify possibly immeasurably small changes in the transcriptome and the proteome, even when derived from minor changes in the genome [82–84]. Note here that in metabolic networks the parameters are typically the starting enzyme concentrations and rate constants, whereas the system variables are the metabolic fluxes and concentrations, and that as in all systems the parameters control the variables and not vice versa. This recognition that small changes in network parameters can cause large changes in metabolite concentrations has led to the concept of metabolites as biomarkers for diseases. Although an important topic, it has been reviewed multiple times recently [85–105] and, for reasons of space and the rarity of their assessment via network biology, disease biomarkers are not our focus here.

## Modelling biochemical networks – how we do so

Although one could seek to understand the time-dependent spatial distribution of signalling and metabolic substances within individual cellular compartments [106,107] and while spatially discriminating analytical methods such as Raman spectroscopy [108] and mass spectrometry [109–113] do exist for the analysis of drugs *in situ*, the commonest type of modelling, as in the spread of substances in ecosystems [114], assumes ‘fully mixed’ compartments and thus ‘pools’ of metabolites, cf. [115,116]. Although an approximation, this ‘bulk’ modelling will be necessary for complex ecosystems such as humans where, in addition to the need for tissue- and cell-specific models, microbial communities inhabit this superorganism and the gut serves as a source for nutrients courtesy of these symbionts [117]. The gut microflora contain some 10^13^–10^14^ bacteria (over 1000 bacterial species, each with their own unique metabolic network) that allow metabolite transformation and cross-feeding within the prokaryotic group and to our gut epithelia; it is also noteworthy that, although antibiotics have an obvious effect here, other human-targeted pharmaceuticals will also undergo microbial drug transformation [117] and cause shifts in gut flora metabolism [118]. Overall, metabolites can be seen as the nodes of (mathematical) graphs [119] – familiar as the conventional biochemical networks of laboratory posters [120], now available digitally – for which the edges reflect enzymes catalysing interconversions of biochemical substances (as well as transporters, see below). Modelling such networks typically involves a four-stage approach [19,20,53,54,121].

In the first, qualitative stage we list all the reactions that are known to occur in the organism or system of interest. It is increasingly possible to automate this [122–126], including through the use of the techniques of text mining [127–131]. A second stage, also qualitative, adds known effectors (activators and inhibitors). The third and fourth stages are more quantitative in character and involve addition of the known, or surrogate [132–134], kinetic rate equations and the values of their parameters (such as *K*_cat_ and *K*_*m*_). Given such information, it is then possible to provide a stochastic [135,136] or ordinary [137] differential equation model of the entire metabolic network of interest, typically encoded in the Systems Biology Markup Language (SBML; http://sbml.org/) [138], using one of the many suites of software available, for example Cell Designer [139], COPASI [140–143] or Cytoscape [144,145].

## Topology and stoichiometry of metabolic networks as major constraints on fluxes

Given their topology, which admits a wide range of parameters for delivering the same output effects and thereby reflects biological robustness [146–149], metabolic networks have two especially important constraints that assist their accurate modelling [58,77,150,151]: (i) the conservation of mass and charge, and (ii) stoichiometric and thermodynamic constraints [152]. These are tighter constraints than apply to signalling networks.

## New developments in modelling the human metabolic network

Since 2007 [153,154], several groups have been developing improved but nonidentical [155] models of the human metabolic network at a generalised level [156–159] and in tissue-specific [160–168] forms. Following a similar community-driven [169] strategy in *Saccharomyces cerevisiae* [121], surprisingly similar to humans [170,171], and in *Salmonella typhimurium* [172], we focus in particular on a recent consensus paper [159] that provides a highly curated and semantically annotated [55,173,174] model of the human metabolic network, termed Recon2 (http://humanmetabolism.org/). In this work [159], a substantial number of the major groups active in this area came together to provide a carefully and manually constructed/curated network, consisting of some 1789 enzyme-encoding genes, 7440 reactions and 2626 unique metabolites distributed over eight cellular compartments. Note, however, that a variety of dead-end metabolites and blocked reactions remain (essentially orphans and widows). Nevertheless, Recon2 was able to account for some 235 inborn errors of metabolism, see also [175], as well as a huge variety of metabolic ‘tasks’ (defined as a non-zero flux through a reaction or through a pathway leading to the production of a metabolite Q from a metabolite P). In addition, filtering based on expression profiling allowed the constrution of 65 cell-type-specific models. Excreted or exometabolites [176–182] are a particularly interesting set of metabolites, and Recon2 could predict successfully a substantial fraction of those [159].

## Role of transporters in metabolic fluxes

**Figure 3:**
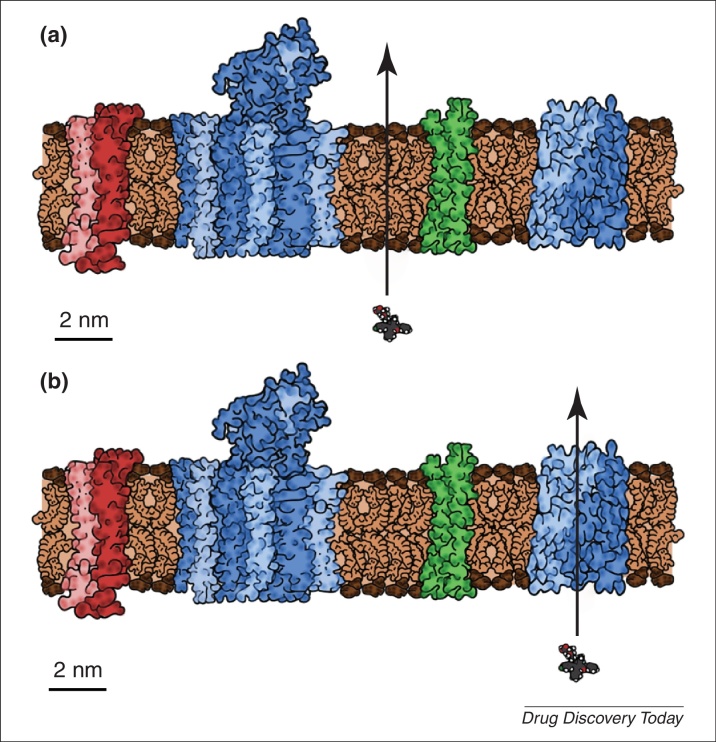
Two views of the role of solute carriers and other transporters in cellular drug uptake. **(a)** A more traditional view in which all so-called ‘passive’ drug uptake occurs through any unperturbed bilayer portion of membrane that might be present. **(b)** A view in which the overwhelming fraction of drug is taken up via solute transporters or other carriers that are normally used for the uptake of intermediary metabolites. Noting that the protein:lipid ratio of biomembranes is typically 3:1 to 1:1 and that proteins vary in mass and density [440,441] (a typical density is 1.37 g/ml [441]) as does their extension, for example, see [442], normal to the ca. 4.5 nm [443] lipid bilayer region, the figure attempts to portray a section of a membrane with realistic or typical sizes [441] and amounts of proteins and lipids. Typical protein areas when viewed normal to the membrane are 30% [444,445], membranes are rather more ‘mosaic’ than ‘fluid’ [442,446] and there is some evidence that there might be no genuinely ‘free’ bulk lipids (typical phospholipid masses are ∼750 Da) in biomembranes that are uninfluenced by proteins [447]. Also shown is a typical drug: atorvastatin (Lipitor^®^) – with a molecular mass of 558.64 Da – for size comparison purposes. If proteins are modelled as cylinders, a cylinder with a diameter of 3.6 nm and a length of 6 nm has a molecular mass of ca. 50 kDa. Note of course that in a ‘static’ picture we cannot show the dynamics of either phospholipid chains (e.g. [448]) or lipid (e.g. [449–451]) or protein diffusion (e.g. [452,453]).

The uptake and excretion of metabolites between cells and their macrocompartments requires specific transporters and in the order of one third of ‘metabolic’ enzymes [153,154], and indeed of membrane proteins [183,184], are in fact transporters or equivalent. What is of particular interest (to drug discovery), based on their structural similarities [185–188], is the increasing recognition [149,189–199] (Fig. 3) that pharmaceutical drugs also get into and out of cells by ‘hitchhiking’ on such transporters, and not – to any significant extent – by passing through phospholipid bilayer portions of cellular membranes. This makes drug discovery even more a problem of systems biology than of biophysics.

## ‘Newly discovered’ metabolites and/or their roles

To illustrate the ‘unfinished’ nature even of Recon2, which concentrates on the metabolites created via enzymes encoded in the human genome, and leaving aside the more exotic metabolites of drugs and foodstuffs and the ‘secondary’ [200] metabolites of microorganisms, there are several examples of interesting ‘new’ (i.e. more or less recently recognised) human metabolites or roles thereof that are worth highlighting, often from studies seeking biomarkers of various diseases – for caveats of biomarker discovery, which is not a topic that we are covering here, and the need for appropriate experimental design, see [201]. Examples include *N*-acetyltaurine [202], 27-nor-5β-cholestane-3,7,12,24,25 pentol glucuronide [203], the cytidine-5-monophosphate:pentadecanoic acid ratio [204], desmosterol [205], F_2_-isoprostanes [206–208], galactose-6-phosphate [209], globotriaosylsphingosines (lyso-Gb3) [210,211], cyclic GMP-AMP [212,213], hexacosanedioic acid [214], l-homoarginine [94,215,216], d-2-hydroxyglutarate [217,218], 3-(4-hydroxy-phenyl)propionic acid [219], 3-methyl histidine [220], 3-indoxyl sulphate [221], *N*-methyl nicotinamide [188,222], neopterin [223–225], ophthalmic acid [226], *O*-phosphoethanolamine [227], 2-piperidinone [228], pseudouridine [229], 4-pyridone-3-carboxamide-1-β-d-ribonucleoside triphosphate [230], Se-methylselenoneine [231], a mammalian siderophore [232–234], sphinganine [235], sphingosine-1-phosphate [236], succinyltaurine [237] and 3-ureido-propionate [238], as well as a variety of metabolites coming from or modulated by the human microbiome [100,117,239–244]. Other classes of metabolites not well represented in Recon2 are oxidised molecules [245] such as those caused by nonenzymatic reaction of metabolites with free radicals such as the hydroxyl radical generated by unliganded iron [246–250]. There is also significant interest in using methods of determining small molecules such as those in the metabolome (*inter alia*) for assessing the ‘exposome’ [251–255], in other words all the potentially polluting agents to which an individual has been exposed [256].

## Recently discovered effects of metabolites on enzymes

Another combinatorial problem [61] reflects the fact that in molecular enzymology it is not normally realistic to assess every possible metabolite to determine whether it is an effector (i.e. activator or inhibitor) of the enzyme under study. Typical proteins are highly promiscuous [199,257,258] and there is increasing evidence for the comparative promiscuity of metabolites [259–261] and pharmaceutical drugs [26,39,199,262–271]. Certainly the contribution of individual small effects of multiple parameter changes can have substantial effects on the potential flux through an overall pathway [272], which makes ‘bottom up’ modelling an inexact science [273]. Even merely mimicking the *in vivo* (in *Escherichia coli*) concentrations of K^+^, Na^+^, Mg^2+^, phosphate, glutamate, sulphate and Cl^−^ significantly modulated the activities of several enzymes tested relative to the ‘usual’ assay conditions [274]. Consequently, we need to be alive to the possibility of many (potentially major) interactions of which we are as yet ignorant. One class of example relates to the effects of the very widespread [275] post-translational modification on metabolic enzyme activities. Other recent examples include ‘unexpected’ effects of β-hydroxybutyrate on histone deacetylase [276], of serine on pyruvate kinase [277], of threonine on histone methylation and stem cell fate [278], of trehalose-6-phosphate on plant flowering time [279] and of lauroyl carnitine on macrophages [280].

In addition, some metabolites are known to affect drug transportation into cells; a well known example of this occurs with grapefruit [281–285], which contains naringin [286] that in humans is metabolised to naringenin [287]. As well as interacting with transporters to change absorption of drugs across the gut which modulates their bioavailability, these phytochemicals also inhibit various P450 activities and this can lead to prolonged and elevated drug levels; indeed several deaths have been linked to the consumption of grapefruit altering the concentration and/or bioavailability of a variety of pharmaceuticals.

## Constraint-based modelling of metabolic fluxes

**Figure 4:**
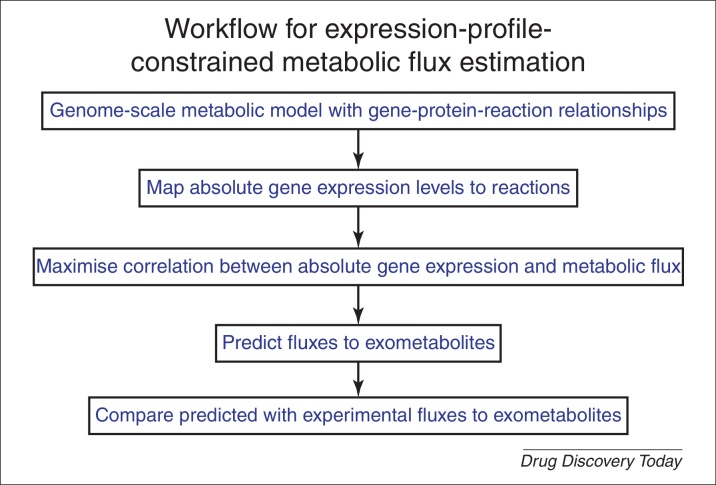
The steps in a workflow that uses constraints based on (i) metabolic network stoichiometry and chemical reaction properties (both encoded in the model) plus, and (ii) absolute (RNA-Seq) transcript expression profiles to enable the accurate modelling of pathway and exometabolite fluxes. The full strategy and results are described in [297].

Armed with the metabolic network models, it is possible to predict metabolic fluxes directly. This can be done in a ‘forward’ direction (as above; given the network, starting concentrations of enzymes and metabolites, and rate equations one can then predict the fluxes), in an ‘inverse’ direction (given the fluxes and concentrations one can try to predict the enzyme concentrations and kinetic parameters that would account for them [288–296]) or iteratively, using both kinds of knowledge. Historically, it has been common to use a ‘biomass’ term as a kind of dumping ground for uncertain fluxes. However, a recent and important discovery [297] (Fig. 4) is that a single transcriptome experiment, serving as a surrogate for fluxes through individual steps, provides a huge constraint on possible models, and predicts in a numerically tractable way and with much improved accuracy the fluxes to exometabolites without the need for such a variable ‘biomass’ term. Other recent and related strategies that exploit modern advances in ’omics and network biology to limit the search space in constraint-based metabolic modelling include references [137,151,298–306].

## Improvements in methods for measuring metabolites

Since its modern beginnings [78,307–310], metabolomics is significantly seen as an analytical science, in that it depends on our ability to measure sensitively, precisely and accurately the concentrations of a multitude of chemically diverse metabolites. As such it is worth highlighting a few recent papers that have improved these abilities – mainly via improvements in chromatography–mass spectrometry [81,84,102,311–322] in terms of increased coverage [255,323–329], metabolite identification [316,330–341], flux and pathway analysis [65,301,342–354], long-term robustness [355,356], sensitivity [357–359], precision [315,358,360–364], discrimination [228,287,365–367], among others. It is clear from the above that many analytical approaches are used to measure metabolites and, in addition to the chemical diversity of metabolites, each metabolomics platform typically has different levels of sensitivity. NMR spectroscopy measures small molecules typically in the μm to high mm range, gas chromatography–mass spectrometry (GC–MS) detects metabolites in the range from μm to mm and liquid chromatography (LC)–MS significantly lower in the nm to μm levels [368]. Sample preparation is also an important and sometimes overlooked component of the analysis [369,370], and can be based on predictable chemistry [371].

Novel methods of data analysis also remain very important [372,373], and some examples of these include metabolomics pipelines [374,375], peak alignment [376] and calibration transfer [377–379], between-metabolite relationships [380], metabolite time series comparisons [381], cross correlations [382], multiblock principal components [383] and partial least squares [384] analysis, metabolome databases [340,385–396], methods for mode-of-action discovery [365,397–401], data management [402,403] and standards [404,405], and statistical robustness [406,407].

## Concluding remarks – the role of metabolomics in systems pharmacology

What is becoming increasingly clear, as we recognise that to understand living organisms in health and disease we must treat them as systems [96,149], is that we must bring together our knowledge of the topologies and kinetics of metabolic networks with our knowledge of the metabolite concentrations (i.e. metabolomes) and fluxes. Because of the huge constraints imposed on metabolism by reaction stoichiometries, mass conservation and thermodynamics, comparatively few well-chosen ’omics measurements might be needed to do this reliably [297] (Fig. 4). Indeed, a similar approach exploiting constraints has come to the fore in *de novo* protein folding and interaction studies [408–412].

What this leads us to in drug discovery is the need to develop and exploit a ‘systems pharmacology’ [18,30,32,40,45–47,149,156,413–429] where multiple binding targets are chosen purposely and simultaneously. Along with other measures such as phenotypic screening [8,430,431], and the integrating of the full suite of e-science approaches [44,131,405,432–439], one can anticipate considerable improvements in the rate of discovery of safe and effective drugs.
