# Corrigendum to “Metabolomics and systems pharmacology: why and how to model the human metabolic network for drug discovery” [Drug Discov. Today 19 (2014), 171–182^☆^

**DOI:** 10.1016/j.drudis.2014.03.019

**Published:** 2014-11

**Authors:** Douglas B Kell, Royston Goodacre

**Affiliations:** School of Chemistry and Manchester Institute of Biotechnology, The University of Manchester, 131 Princess Street, Manchester M1 7DN, UK

Due to our error, the article has been published with the incorrect Open Access License. The correct one is:

This is an open-access article distributed under the terms of the of the Creative Commons Attribution License, which permits unrestricted use, distribution and reproduction in any medium, provided the original author and source are credited.

We offer our sincere apologies to the Authors and our Readership for this occurrence.

